# Artificial intelligence in acute and critical care: current challenges and strategic solutions

**DOI:** 10.3389/fpubh.2026.1818726

**Published:** 2026-04-10

**Authors:** Tianle Gao, Tingting Fan, Wenying Leng, Shenghui Yu, Qingguo Lyu

**Affiliations:** 1 Emergency Center, Chengdu Integrated TCM&Western Medicine Hospital, Chengdu, China; 2 Department of Endocrinology and Metabolism, West China Hospital, Sichuan University, Chengdu, China

**Keywords:** acute and critical illnesses, artificial intelligence, data quality, decision support, ethical challenges

## Abstract

**Background:**

The Emergency Department (ED) and Intensive Care Unit (ICU) are high-acuity environments where rapid decision-making and clinical precision are fundamental to patient survival. Artificial Intelligence (AI) offers transformative potential by providing real-time data synthesis, advanced pattern recognition, and personalized decision support—capabilities essential for optimizing clinical efficiency and patient outcomes. This narrative review synthesized the applications, clinical benefits, and implementation challenges of AI within acute and critical care.

**Methods:**

A comprehensive literature search was conducted across international and Chinese databases, including PubMed, Web of Science, China National Knowledge Infrastructure (CNKI), and Wanfang Data, yielding 281 English and 136 Chinese studies. Furthermore, official policy documents from provincial Health Commissions were analyzed to evaluate the regulatory landscape for AI deployment.

**Results:**

AI showed potential across multiple acute and critical care scenarios, including early warning of sepsis, chest pain assessment, stroke imaging, electrocardiogram interpretation for acute coronary syndrome, ARDS subphenotyping, cardiogenic shock risk stratification, treatment support, and workflow coordination. However, translation into real-world practice remained limited by major challenges, including poor data quality, heterogeneity and fragmentation of clinical data, limited model explainability, insufficient prospective validation, workflow integration barriers, clinician training gaps, and unresolved ethical and regulatory issues.

**Conclusions:**

Medical AI held substantial promise for improving decision-making efficiency, workflow optimization, and patient outcomes in acute and critical care. However, its safe and effective implementation required more than predictive performance alone. Future progress depended on trustworthy, explainable, workflow-integrated, and prospectively validated systems supported by stronger data infrastructure, interdisciplinary collaboration, and clearer ethical and regulatory oversight. This review proposed a translational framework and a general workflow for data-driven AI development in acute and critical care.

## 1 Introduction

Acute and critical illnesses posed an immediate and substantial threat to patient survival due to their inherent complexity, heterogeneity, and unpredictability (1). These conditions encompassed a broad spectrum of diseases, each characterized by intricate pathophysiological mechanisms that often involve multiple organ systems and dynamic physiological alterations (2). The emergency department (ED) and intensive care unit (ICU) are environments where rapid decision-making and precision are vital for patient survival. These settings are defined by their fast pace, high patient turnover, unpredictable workloads, and the constant management of acute, life-threatening conditions (3).

The healthcare field underwent a profound digital transformation driven by rapid advances in information technology. Artificial intelligence (AI), big data, and cloud computing integrated into medical practice, reshaping diagnostic and therapeutic paradigms (4). Since 2023, the emergence of generative AI accelerated this transition, opening new avenues for acute and critical care (5). AI holds transformative potential by enabling real-time data analysis, pattern recognition, and personalized decision support—capabilities essential for optimizing clinical workflows and improving patient outcomes. Large language models (LLMs) demonstrated unprecedented ability to process not only structured data but also unstructured sources such as clinical notes and imaging reports (6). Their multimodal integration capabilities position them as powerful tools in complex clinical environments (6). By simulating aspects of human clinical reasoning, AI-enabled systems provided timely and accurate assessment of critically ill patients, potentially improving outcomes (7). Generative approaches leveraged self-attention mechanisms to capture cross-modal interactions, a feature aligned with the temporal density, fragmented information, and high-stakes decision-making characteristic of emergency care (8).

Unlike previous reviews that had mainly summarized AI applications or technical performance in critical care, this review adopted a translational framework centered on decision-making in acute and critical care. Specifically, it linked common AI use cases to bedside actions in emergency departments and ICUs, examined how data, workflow, and governance challenges emerged in time-sensitive clinical environments, and outlined a pathway from predictive performance to real-world clinical utility. By integrating clinical use cases, implementation barriers, regulatory comparison, and causal evaluation strategies, the review aimed to clarify both the opportunities and limitations of AI in acute and critical care and to identify priorities for future research.

## 2 Method

This study was conducted as a narrative review. A comprehensive literature search was conducted in both English and Chinese databases, including PubMed, Web of Science, CNKI, and Wanfang Data. Search terms related to AI technologies and clinical settings (e.g., “emergency medicine,” “intensive care,” “Intensive Care Unit,” “ICU,” “critical care,” and “acute care”) were searched within titles and abstracts. The search covered articles published between January 1, 2021, and January 09, 2026. The main search string used was as follows: [“artificial intelligence”(Title/Abstract) OR “machine learning”(Title/Abstract) OR “deep learning”(Title/Abstract) OR “large language model^*^”(Title/Abstract) OR “generative AI”(Title/Abstract)] AND [“critical care”(Title/Abstract) OR “intensive care”(Title/Abstract) OR “ICU”(Title/Abstract) OR “emergency medicine”(Title/Abstract) OR “acute care”(Title/Abstract)].

Studies were included if they met the following criteria:

1. focused on the application, evaluation, or implementation of AI technologies in acute or critical care settings;
2. involved clinical data or clinical decision-support scenarios in emergency departments or intensive care units;
3. were original research articles, systematic reviews, scoping reviews, or narrative reviews; and
4. were published in English or Chinese.

**Figure 1:**
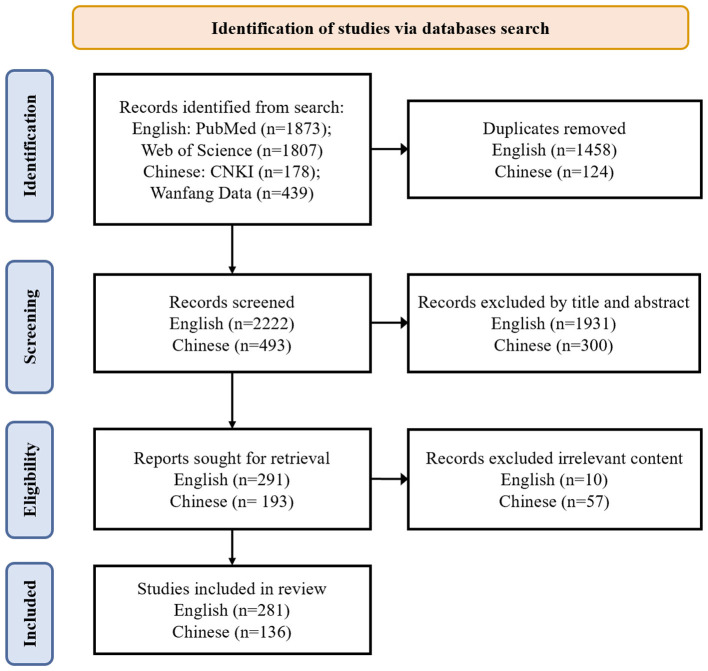
Flow diagram depicting the strategy for literature search and selection.

Studies were excluded if they were editorials, commentaries, conference abstracts without sufficient methodological details, animal studies, or studies unrelated to acute or critical care medicine. After removing duplicates, titles and abstracts were screened for relevance. Full texts of potentially eligible articles were subsequently reviewed to determine final inclusion. Study screening was independently performed by two reviewers. Any disagreements regarding study eligibility were resolved through discussion, and when necessary, a third reviewer was consulted to reach consensus. The details of the literature search were presented in Figure 1. Because this was a narrative review, formal risk-of-bias scoring was not performed. Instead, the included literature was synthesized thematically according to four predefined domains: clinical use cases, data-related challenges, implementation barriers, and ethical/regulatory considerations.

To identify relevant national policies and directives, we systematically searched official websites of the National Health Commission of the People's Republic of China. These websites represent the authoritative source of healthcare policies in China. We focused on documents published between January 2021 and December 20, 2025, corresponding to the period of major policy reforms in AI. We used advanced search functionalities with keywords such as “artificial intelligence,” “medical big data,” “digital health,” and “Internet + healthcare.” Policy documents unrelated to medical AI application or lacking clear directives on clinical implementation, data governance, or ethical standards were excluded. For international comparison, we additionally reviewed two major regulatory documents on medical AI: Regulation (EU) 2024/1689 (the European Union Artificial Intelligence Act, EU AI Act) (9) and the U.S. Food and Drug Administration draft guidance Artificial Intelligence-Enabled Device Software Functions: Lifecycle Management and Marketing Submission Recommendations (January 2025) (10).

## 3 Results

A total of 4,297 records were identified through database searching, including 1,873 from PubMed, 1,807 from Web of Science, 178 from CNKI, and 439 from Wanfang Data. After duplicate removal, 2,715 records (2,222 English-language and 493 Chinese-language) were screened by title and abstract. Full texts were retrieved for 484 reports, of which 67 were excluded because of insufficient relevance to the review topic. Ultimately, 417 studies were included in the final review, comprising 281 English-language and 136 Chinese-language publications. Formal quality assessment was not conducted because of the narrative design of the review and the heterogeneity of included evidence types.

To provide a focused synthesis, this section is organized around a translational framework that links three questions: where AI is being applied in acute and critical care, why many systems remain difficult to deploy safely at the bedside, and how future research can bridge the gap between algorithmic performance and clinically meaningful benefit.

### 3.1 Workflow integration in acute and critical care

The ED and ICU shared several core challenges in AI workflow integration (11). Both settings were high-acuity, time-sensitive, and data-intensive environments, so AI tools needed to fit existing clinical workflows rather than operate as isolated systems (11, 12). Implementation depended on interoperable digital infrastructure, prospective validation, clinician education, and ongoing oversight after deployment. More broadly, recent consensus guidance emphasized that trustworthy healthcare AI required lifecycle planning, including usability, traceability, robustness, explainability, and post-deployment monitoring, rather than model development alone (13).

Despite these shared requirements, workflow barriers differ between the ED and the ICU. In the ED, AI has more commonly been studied for front-end operational and decision-support tasks, such as triage, wait-time prediction, crowding management, patient flow, and early identification of high-risk presentations (14). Accordingly, implementation in the ED depends heavily on real-time workflow compatibility, rapid usability, and local validation under conditions of frequent interruptions and time pressure (13). ICU implementation places greater emphasis on continuous multimodal data integration, repeated risk reassessment over time, and coordination with bedside monitoring, electronic health records, and decision-support platforms (11).

### 3.2 Clinical use cases of AI in acute and critical care

#### 3.2.1 Triage and early deterioration detection

In acute and critical care, sepsis early warning is a representative application of AI for triage and early deterioration detection. By integrating electronic health records (EHRs), serial vital signs, laboratory results, medication records, and nursing documentation, machine learning (ML) models can identify patients at risk of sepsis before overt clinical deterioration becomes apparent, thereby enabling earlier antimicrobial therapy, fluid resuscitation, escalation of monitoring, and senior clinical review (15–17).

Several studies suggest that AI-based sepsis warning systems can outperform conventional screening tools in selected settings, although performance varies substantially depending on the model, population, and alert threshold. Giannini et al. (18) developed a random forest model with high specificity (98%) but low sensitivity (26%), highlighting the trade-off between reducing false positives and missing true cases. Calvert et al. (19) showed that a model based on only six vital signs could predict sepsis up to 48 h before onset, with better performance than traditional scores. Yang et al. (20) reported an explainable model with an area under the curve (AUC) of 0.85 and a sensitivity of 90%, although specificity remained modest at 64%. Shashikumar et al. (21) further demonstrated that Deep Artificial Intelligence Sepsis Expert (DeepAISE) could predict sepsis several hours in advance while also identifying patient-specific risk factors in real time.

#### 3.2.2 AI-assisted chest pain assessment

Recent studies have also explored the use of large language models (LLMs) as clinical decision-support tools in chest pain assessment. In a randomized controlled trial involving 50 physicians reviewing standardized chest pain cases (22), physicians modified their clinical decisions after receiving GPT-4–generated recommendations, resulting in more accurate clinical decisions. In a study of 2,424 ED cases, an AI-based triage model achieved an overall accuracy of 0.96 and a macro-average F1-score of 0.94 (23), demonstrating balanced performance across cardiac and non-cardiac risk groups. These findings suggest that combining clinical and linguistic features may improve early risk stratification and facilitate faster disposition decisions for patients presenting with chest pain in the ED.

#### 3.2.3 Diagnosis and classification

AI had increased been applied to computed tomography (CT) imaging to support rapid diagnosis and classification of acute stroke (24). Deep learning models, particularly convolutional neural networks (CNNs), were able to automatically detect intracranial hemorrhage, large-vessel occlusion, and early ischemic changes on CT scans. Several FDA-cleared platforms, including Viz.ai, RapidAI, and e-ASPECTS, analyzed CT or CT angiography images and generated automated alerts for suspected stroke lesions (24–26). For example, Viz.ai detected large-vessel occlusions on CT angiography with approximately 78% sensitivity and 97% specificity, enabling earlier notification of stroke teams and facilitating faster treatment decisions (27). By accelerating image interpretation and standardizing lesion assessment, AI-assisted CT analysis improved diagnostic efficiency in acute stroke care.

AI-assisted electrocardiogram (ECG) interpretation had considerable potential in the early detection and triage of acute coronary syndrome (ACS). The 12-lead ECG remained a cornerstone of first-line assessment for myocardial ischemia and myocardial infarction, but its interpretation was challenging and showed variability among clinicians (28). Deep learning models, especially convolutional neural networks (CNNs), demonstrated the ability to analyze ECG data and identify myocardial infarction and other cardiac abnormalities with high diagnostic performance (29). In several studies, reported diagnostic accuracy exceeded 90%, although these findings depended on study design and validation conditions (30). Overall, AI-assisted ECG interpretation appeared to provide useful support for rapid clinical decision-making, especially in settings with limited access to specialist cardiology expertise (29).

AI has increasingly been applied to identify phenotypes of acute respiratory distress syndrome (ARDS), a syndrome characterized by marked clinical and biological heterogeneity. Machine learning algorithms can integrate multidimensional ICU data to identify distinct patient subgroups. For instance, Calfee et al. (31) analyzed data from two randomized clinical trials including over 1,000 ARDS patients and identified hyperinflammatory and hypoinflammatory subphenotypes, with mortality rates of approximately 45 and 20%, respectively. Similarly, Maddali et al. (32) validated machine learning–derived ARDS subphenotypes across multiple observational cohorts, demonstrating consistent differences in inflammatory markers and clinical outcomes. AI-driven phenotype recognition may improve risk stratification and support precision treatment strategies in ARDS.

#### 3.2.4 Dynamic risk stratification for cardiogenic shock

AI-based trajectory forecasting models have been explored for predicting deterioration in patients at risk of cardiogenic shock. By integrating longitudinal clinical variables such as vital signs, laboratory parameters, and electrocardiographic features, machine learning algorithms can dynamically assess the progression from ST-elevation myocardial infarction (STEMI) to cardiogenic shock. For example, Stamate et al. (33) developed phase-specific machine learning models using real-world clinical data from 2,856 patients with acute coronary syndrome, including 158 STEMI patients with cardiogenic shock, to predict shock progression across prehospital and emergency care stages. Their results showed that the Extra Trees model achieved the highest performance in the prehospital phase with an accuracy of 90.62%, while Random Forest reached 81.25% accuracy during cardiology consultation, demonstrating the potential of AI models for early deterioration prediction and dynamic risk stratification in cardiogenic shock. These approaches may support earlier recognition and intervention, although prospective validation is still needed to determine whether their use improves patient outcomes (34).

By combining arrest-related information, physiologic data, neurologic findings, and biomarkers, machine learning models may help estimate the risk of poor neurologic outcome after return of spontaneous circulation (ROSC) (35). These tools may support early risk stratification, but they should be used only as adjuncts to guideline-based multimodal assessment rather than as a standalone basis for treatment decisions (35, 36).

#### 3.2.5 AI-assisted treatment support

Beyond early detection, AI has also been evaluated for its potential to support treatment decisions and improve the timeliness of therapeutic interventions in sepsis. Burdick et al. (37) found that use of an ML-based sepsis prediction algorithm was associated with lower in-hospital mortality, shorter hospital stay, and fewer 30-day readmissions. In the emergency department (ED), Kijpaisalratana et al. (38) showed that a real-time ML-assisted sepsis alert improved the timeliness of antibiotic administration. Adams et al. (39) reported that the Targeted Real-Time Early Warning System (TREWS) was associated with a 1.85-h reduction in median time to first antibiotic order and with improved outcomes after adjustment for illness severity.

AI has also been applied to predict clinical deterioration and guide respiratory support strategies in critically ill patients. Lu et al. (40) proposed a hybrid framework combining a counterfactual inference model with a LLM to guide respiratory support decisions between high-flow nasal cannula (HFNC) and non-invasive ventilation (NIV). In 1,261 ICU encounters, treatment decisions concordant with LLM-enhanced recommendations were associated with lower invasive mechanical ventilation rates and reduced mortality risk, highlighting the potential of AI-assisted decision support for individualized respiratory management in critical care (40).

#### 3.2.6 Operational workflow support

AI also supported workflow coordination across the acute-care pathway. This was especially relevant in prehospital transport, emergency department operations, and triage support. In the prehospital setting, Kim et al. (41) evaluated the CONNECT-AI platform. This system combined real-time patient data with hospital resource information, such as bed occupancy and procedural availability. Although median transport times were similar between groups, the system reduced transport outliers among patients with fever or respiratory symptoms and was associated with lower mortality (41). These findings suggested that AI improved destination selection by matching ambulance routing to real-time hospital capacity.

Within the hospital, AI-based forecasting models also helped predict ED crowding and improve staffing allocation. Akbasli et al. (42) developed and compared 20 forecasting models using data from 352,843 pediatric ED visits. Their results showed that advanced deep learning models performed better than traditional models. These models also improved physician allocation during peak hours. In addition, they reduced the patient-to-physician ratio (42). However, the role of AI in triage remained limited. Although AI-based triage systems seemed promising, retrospective studies showed that human triage still performed better for key outcomes, such as mortality and life-saving interventions (43, 44). Therefore, the main value of AI in workflow coordination was to support crowding management, staffing, and patient flow, rather than to replace clinician-led triage (44).

Table 1 provides illustrative examples of how AI tools may support bedside decision-making in acute and critical care. The listed scenarios are intended to highlight clinically relevant use cases, typical data inputs, and potential modes of clinician interaction.

**Table 1:** Clinically specific examples of AI support in acute and critical care.

Scenario	Typical inputs	What AI can add	How clinicians interact with the tool
ED chest pain and suspected acute coronary syndrome (22, 23, 28–30)	Symptoms, serial ECGs, troponin values, vitals, prior coronary history, medication use	Rapid ECG interpretation, dynamic rule-in and rule-out support, prioritization for cardiology review or immediate cath lab activation	Embedded within a chest-pain pathway; the ED physician reviews the risk estimate together with the ECG trace, troponin trend, and the top contributing findings before acting
Cardiogenic shock in the ED, CICU, or ICU (33, 34)	Mean arterial pressure, lactate, urine output, vasoactive dose, bedside echo, mechanical support data, organ-failure markers	Early detection of hemodynamic collapse, trajectory forecasting, and prompts for escalation, reassessment, or shock-team activation	Displayed as a time-series dashboard that is reviewed during bedside reassessment and multidisciplinary huddles rather than as a standalone static score
Cardiac arrest and post-resuscitation care (121)	Rhythm strips, compression depth and rate, no-flow and low-flow time, end-tidal CO2, postarrest neurologic exam and biomarkers	Rhythm classification, feedback on CPR quality, postarrest prognostic support, and identification of patients requiring rapid escalation	Real-time feedback may support the code team, while post-ROSC outputs should be reviewed by a multidisciplinary team using guideline-based multimodal assessment
Sepsis and occult deterioration (15–21, 37–39)	Vitals, nursing observations, laboratory trends, microbiology, notes, fluid balance, antibiotic timing	Earlier recognition of deterioration, risk trajectories, and prioritization of time-sensitive bundle elements	Alerts are routed to the primary team together with trend plots and the expected response window so that the model points to action rather than merely prediction
Acute respiratory failure and ARDS (31, 32, 40)	Waveforms, ventilator settings, arterial blood gases, imaging reports, inflammatory markers, sedation status	Support for phenotype recognition, respiratory deterioration detection, and reassessment of ventilation or proning strategy	The ICU team reviews waveform and blood-gas trends together with the AI output during repeated bedside evaluation
Stroke or trauma triage (24–27, 41)	Prehospital observations, triage notes, point-of-care imaging, neurologic examination, vital signs	Faster image or signal classification and prioritization for transfer, thrombolysis, thrombectomy, or trauma-team activation	Used as triage support to accelerate review and communication, not as a replacement for specialist interpretation

AI, artificial intelligence; ACS, acute coronary syndrome; ARDS, acute respiratory distress syndrome; CICU, cardiac intensive care unit; CPR, cardiopulmonary resuscitation; ECG, electrocardiogram; ED, emergency department; ICU, intensive care unit; ROSC, return of spontaneous circulation.

### 3.3 Chinese AI policy

China had been actively developing regulatory frameworks for AI, particularly focusing on generative AI and its application in critical sectors. In June 2021, the General Office of the State Council released the *Opinions on Promoting the High-Quality Development of Public Hospitals*, which emphasized strengthening pre-hospital emergency networks and innovating emergency services (45). Key regulatory milestones include the *Algorithm Recommendation Management Provisions* (2021) (46), the *Deep Synthesis Management Provisions* (2022) (47), the *Interim Measures for the Management of Generative AI Services* (2023) (48), and the *Measures for the Identification of AI-Generated Content* (2025) (49). Collectively, these regulations aimed to promote the ethical and socially responsible use of AI while safeguarding data security, ensuring transparent content labeling, and defining the obligations of service providers. Furthermore, the *Implementation Opinions on Promoting and Regulating the Development of “AI* + *Healthcare” Applications* (2025) explicitly supported the deployment of AI in assisted diagnosis, patient referral services, predictive early warning systems, and the emergency allocation of medical resources (50).

However, several limitations were evident. First, these documents were less explicit in defining how high-risk bedside AI tools should be prospectively validated before use in EDs or ICUs. Second, they provided less detailed guidance on device-level lifecycle oversight, including performance drift, controlled model updating, and post-deployment monitoring of adaptive clinical AI. Third, although they emphasized data governance and algorithm supervision, they were less specific about operational issues that were central to acute and critical care, such as alert fatigue, override mechanisms, local calibration, and documentation of human oversight in time-sensitive clinical decisions.

Regulatory approaches to medical AI differed across China, the European Union, and the United States. Chinese policies mainly emphasized healthcare digitalization, data governance, algorithm supervision, and the incorporation of AI into broader hospital and health-system reform. The EU AI Act established a risk-based legal model, under which healthcare AI was generally treated as high-risk and subject to requirements for risk management, transparency, human oversight, technical documentation, and post-market monitoring (9). In the United States, FDA guidance adopted a product-centered regulatory pathway for AI-enabled medical devices. The framework focused on the safety, effectiveness, validation, and lifecycle management of AI-enabled device software functions, particularly in relation to regulatory submission and clinical use (10).

### 3.4 Data-related challenges

#### 3.4.1 Data quality and reliability

The reliability of medical AI systems was fundamentally dependent on the quality of clinical data (51). Inconsistent or erroneous entries in electronic health records (EHRs) could propagate downstream errors in AI-driven decision-making (52). Such as inconsistent units (e.g., mg/dl vs. mmol/L) in laboratory results, further compromised logical coherence between diagnostic data and clinical interpretation. Moreover, delays in updating EHRs diminished the timeliness of AI-supported decisions, particularly in rapidly deteriorating patients (53). Representativeness was an additional concern. Models trained on datasets that predominantly reflect Western populations could generate inappropriate recommendations for Asian patients, such as misestimation of fluid resuscitation thresholds (54). Data incompleteness, missing values, and poor database architecture exacerbated these challenges, reducing both usability and trustworthiness (55). The temporal and spatial dynamics inherent in critical illness were particularly ill-suited to static datasets, hindering the capacity of generative models to capture rapidly evolving clinical trajectories.

The reliability of AI systems in stroke imaging strongly depends on the quality and diversity of the training datasets. Models trained on limited or biased imaging data may perform well in controlled environments but show reduced accuracy when applied to different patient populations or clinical settings (24, 56). For example, deep learning models developed for stroke detection on CT or MRI require large annotated datasets to accurately identify ischemic lesions or intracranial hemorrhage. However, many neurological datasets remain relatively small or imbalanced, which can reduce model robustness and increase the risk of diagnostic errors in real-world applications (57, 58) (see Table 2).

**Table 2:** Challenges of AI in acute and critical care.

Domain	Key challenges	Illustrative examples
Data-related	Data fragmentation, heterogeneity, and quality issues hinder reliable model training and validation	Incomplete electronic health records; inconsistent imaging protocols; missing outcome data
Technical	Limited model generalizability and interpretability; difficulty in real-time multimodal integration	Black-box nature of deep learning; lack of explainability tools; latency in bedside deployment
Clinical	Insufficient prospective validation and integration into workflow; risk of overreliance on algorithms	Limited multicenter trials; clinician skepticism; alert fatigue in emergency settings
Ethical and legal	Concerns about patient privacy, data security, accountability, and fairness	Ambiguity in liability for AI-driven decisions; potential algorithmic bias in triage or diagnosis
Regulatory	Lack of standardized evaluation frameworks and clear approval pathways for clinical AI tools	Absence of universally accepted performance benchmarks; evolving regulatory requirements

#### 3.4.2 Data standardization

The lack of data interoperability became a hidden bottleneck for AI decision support (55). Heterogeneity in data standards and system architectures limited cross-institutional integration (59). Protocol constraints, such as restricted Fast Healthcare Interoperability Resources (FHIR) interface calls, reduced the efficiency of real-time exchange, while compliance verification under frameworks such as Trusted Exchange Framework and Common Agreement (TEFCA) added further delays (60). In time-sensitive emergencies, fragmented “data silos” posed direct threats to survival, as real-time monitoring, imaging, and ICU parameters remained confined to separate systems. The inability to consolidate these data streams limited multimodal analysis and undermined the value of clinical decision support during the “golden hour.” This isolation also restricted the availability of training samples, forcing reliance on synthetic data and amplifying risks of predictive bias, particularly in rare critical events (61).

#### 3.4.3 Data heterogeneity

Heterogeneity manifested at both structural and conceptual levels (62). Structural heterogeneity referred to differences in data type, format, and attributes, whereas conceptual heterogeneity arose from evolving medical definitions and guidelines. The latter posed unique challenges in critical care. Updates in sepsis criteria from SIRS to SOFA altered biomarker weighting, yet models trained on historical data still applied outdated patterns, causing systematic misclassification (63). Emerging infectious diseases highlighted this vulnerability: COVID-19 variants demonstrated dynamic symptomatology, rendering early predictive models incapable of recognizing atypical presentations such as “silent hypoxemia” (64). The sudden appearance of novel diseases exposed the fragility of generative AI, as models may misapply logic from superficially similar conditions and proposed inappropriate therapies (65).

A key challenge in applying AI to ECG interpretation was data heterogeneity. ECG datasets were often collected from different hospitals, devices, and patient populations, which led to variability in signal characteristics, recording conditions, and waveform features (66). Differences in acquisition systems, signal length, and noise levels required extensive preprocessing and normalization before model training. In addition, dataset imbalance—where normal ECGs were overrepresented compared with pathological cases—affected model performance and reliability (66, 67).

Beyond technical variability, patient-level biological heterogeneity also played an important role. Acute and critical illnesses frequently comprised multiple clinical subphenotypes with distinct physiological or biological profiles. For example, subphenotype analysis in postoperative sepsis demonstrated that patients could be clustered into different clinical groups based on routine physiological and laboratory variables, highlighting substantial heterogeneity within the same disease category (68). Such findings suggested that AI models trained on aggregated populations might perform well on average but fail within specific subgroups, emphasizing the need for subphenotype-aware approaches that stratify patients according to underlying biological characteristics (68).

#### 3.4.4 Model explainability

AI-based recommendations needed to be interpretable to gain clinician trust, particularly in life-or-death scenarios such as multi-organ failure or shock (69). Current deep learning models functioned largely as “black boxes,” offering predictions without transparent reasoning (70). This opacity prevented clinicians from understanding the basis of AI-driven decisions, which limited adoption in high-stakes contexts.

Another important challenge in applying AI to critical care was model explainability. In a recent study, Suñol et al. evaluated LLMs for detecting flow starvation (FS) during mechanical ventilation using 6,500 airway pressure cycles from 28 ICU patients. Although the LLM-generated CNN-1D model achieved high accuracy (0.902, 95% CI 0.899–0.906), the authors emphasized that clinicians may have difficulty interpreting the decision process of AI-generated models (71). The lack of transparent reasoning can reduce clinician trust and hinder adoption in bedside decision-making, highlighting the need for explainable AI frameworks in critical care applications (72).

#### 3.4.5 Model validation

The heterogeneity of critically ill populations complicated large-scale, multicenter trials, while differences in equipment, expertise, and protocols introduced variability in performance outcomes (69). Retrospective testing could not adequately simulate the urgency and resource constraints of real-world emergencies (73). Developing rigorous yet feasible strategies for prospective validation was essential to ensure safety and efficacy in clinical deployment. Many algorithms for stroke diagnosis and outcome prediction had been evaluated primarily using retrospective datasets or single-center studies, which might not accurately reflect real-world clinical conditions (24). For instance, AI systems designed to detect large-vessel occlusions or predict stroke outcomes had demonstrated promising performance in experimental settings but still required large prospective multicenter trials to confirm their reliability and safety in routine clinical practice (24).

#### 3.4.6 Model adaptability

The dynamic evolution of acute and critical illness challenged the adaptability of AI systems, as rare conditions, dataset shift, and emerging disease patterns could outpace model updating and degrade performance over time (74). Without rapid recognition of new trends, AI systems risked misdiagnosis, delayed treatment, and loss of clinical trust, making resilience to low-frequency but high-impact events a critical priority for safe deployment in acute care (75). Critical care required AI systems with exceptional speed and reliability, yet many emergency departments and ICUs lacked the data pipelines, computational capacity, and implementation support needed for real-time deployment (76). In under-resourced settings, hardware and infrastructure limitations further constrained timely deployment during life-threatening events. Prolonged model development, updating, and maintenance demands also slowed iteration, while shortages of interdisciplinary expertise across clinical care, data science, engineering, and informatics limited effective translation into frontline practice (11).

#### 3.4.7 Accuracy and generalizability

Despite promising performance in controlled environments, AI systems often underperformed in real-world acute care settings. Sensitivity and specificity fluctuated widely, undermining clinical trust (77, 78). The heterogeneous nature of critical illness across institutions, patient populations, and comorbidities further limited model generalizability (77, 78). In addition, data inconsistency, incomplete records, and subjective biases in clinical documentation reduced reproducibility and cross-center reliability (79).

These challenges were particularly evident in sepsis early warning systems. Model performance declined across institutions, external validation remained insufficient in many studies, and missing or inaccurate electronic health record (EHR) data affected reliability (20, 21, 80). False-positive alerts contributed to alarm fatigue, overtreatment, and unnecessary antibiotic use (81). Moreover, real-world effectiveness depended heavily on clinician adoption and workflow integration, as provider responses to alerts varied according to workload and clinical context (82). Overall, sepsis early warning illustrated both the potential and the current limitations of AI in acute and critical care triage (37, 83).

#### 3.4.8 Clinician training

A gap persisted between AI development and AI education (84–86). While clinicians possessed deep medical expertise, they lacked systematic training in AI applications, reducing their ability to effectively integrate these tools into acute care decision-making (84, 87). Current curricula rarely incorporated scenario-based AI use, leaving healthcare providers unprepared to leverage real-time feedback during dynamic emergencies (88).

For instance, Ivanov et al. (89) found that a machine-learning-assisted triage system achieved higher accuracy than nurses in assigning Emergency Severity Index levels, but the study did not account for differences in nurse training, clinical experience, or triage education, which might influence real-world performance.

#### 3.4.9 Workflow integration

AI models were frequently developed around accessible datasets rather than the most pressing clinical needs (90). Many tools demonstrated academic novelty but lacked real-world applicability (91). Complex interfaces, poorly integrated outputs, and limited coverage of decision points reduced clinical usability. Most models focused on specific diseases such as stroke or myocardial infarction, neglecting complex syndromes like multi-organ failure or polytrauma (92). This fragmented approach prevented the establishment of a comprehensive decision-support ecosystem. Retrospective studies might not capture the interaction between artificial intelligence systems and clinicians in real clinical practice. Once AI alerted physicians to potential critical outcomes, clinicians might adjust diagnostic testing or interventions, potentially altering patient outcomes and complicating real-world evaluation (93).

#### 3.4.10 Data security and privacy

Healthcare data contained sensitive personal and medical information. Breaches could lead to misuse, psychological harm, and financial consequences, while undermining public trust in data sharing (94). Security capacities varied across institutions, and cross-border data flows in cloud environments raised further risks, complicating regulatory oversight (see Table 1).

AI-based prediction of ED wait times raised ethical and regulatory concerns because these tools could influence patient behavior, staff allocation, and care delivery. Some studies did not fully report preprocessing methods, out-of-sample validation, or subgroup-based performance, which limited confidence in fairness and real-world reliability (95). This issue was important in emergency care, where inaccurate predictions could affect operational decisions. The EU AI Act also highlighted the need for transparency, documentation, human oversight, and safety in high-risk AI systems (9). Data security and privacy were key concerns because most ED wait-time models relied on retrospective electronic health record data and real-time operational variables (95).

#### 3.4.11 Accountability

Current frameworks lacked robust mechanisms to address AI-driven errors. In acute and critical care, where decisions were made within minutes, algorithmic mistakes directly jeopardized survival (11, 96). Establishing clear accountability and remediation pathways was therefore essential to safeguard patient safety and maintain clinician confidence in AI-assisted care (11).

Accountability was particularly challenging in the emergency department and ICU because AI outputs could affect time-sensitive, high-stakes decisions across triage, monitoring, escalation, staffing, and patient flow (11). In these settings, patients' conditions changed rapidly, data came from multiple real-time sources, and decisions were often distributed across several clinicians and digital systems rather than a single actor (76). When an AI-supported decision was delayed, biased, or incorrect, responsibility could be difficult to attribute to one source alone, because performance might depend on the model itself, the input data, the local workflow, the user interface, and the degree of human oversight (97).

### 3.5 Recommendation

#### 3.5.1 From prediction to clinical utility

Future AI studies should move beyond reporting discrimination and instead evaluate whether AI-guided interventions causally improve outcomes under clearly defined and reproducible conditions (98, 99). Many AI models in acute and critical care have shown good predictive performance, but prediction alone does not establish clinical utility. Metrics such as AUROC show how well a model separates higher-risk from lower-risk patients, but they do not show whether acting on model outputs improves outcomes (100).

To address this gap, evaluation should shift from prediction to causal effect estimation. The key question is not whether AI identifies risk accurately, but whether an AI-guided strategy performs better than usual care. Target trial emulation provides a structured way to answer this question using observational data (99, 101). This approach begins by specifying the protocol of the hypothetical randomized trial to be emulated, including eligibility criteria, intervention and comparator strategies, time zero, follow-up, outcomes, estimand, and analysis plan. In AI research, this means defining not only the prediction model but also the clinical action triggered by it, such as escalation of care after an alert (98, 101). The observational analysis should then reproduce that target trial as closely as possible. This is especially important in acute and critical care, where incorrect alignment of eligibility, treatment assignment, and follow-up can introduce substantial bias, including immortal time bias and time-varying confounding (102, 103).

Depending on the clinical question and data structure, analyses may use inverse probability weighting, g-methods, instrumental variables, or difference-in-differences approaches. These methods are useful when treatment decisions evolve over time and depend on changing patient status (98).

#### 3.5.2 Improving data quality

Constructing high-quality medical information systems formed the foundation of reliable data quality (104). Optimizing system architecture and adding AI-assisted data validation and error correction functions could reduce input errors from the technical side (105). In parallel, strengthening clinician training and standardizing data entry procedures improved accuracy and consistency (106, 107). Comprehensive quality management frameworks encompassed data collection, storage, transmission, and analysis. Regular audits and monitoring helped identify and correct data errors in a timely manner. AI and big data technologies were applied to automatic cleaning, deduplication, and correction to enhance accuracy and consistency. For emergency and ICU settings, Artificial Intelligence of Things (AIoT) tailored to physiologic models was developed to address inevitable missing or abnormal values during resuscitation (108). This approach relied not only on statistical models but also incorporated critical care pathophysiology to ensure clinical plausibility. LLMs extracted key information from unstructured sources such as medical records or imaging reports, converting them into structured features for downstream ML models (109).

#### 3.5.3 Standardization and heterogeneity in data

Promoting unified clinical data standards was essential for improving data quality in acute and critical care. Mandatory adoption of standardized formats and coding, especially for vital signs, severity scores, interventions, and outcome measures, could reduce inconsistency across emergency departments, ICUs, and prehospital systems. Data collection standards could also be tiered according to institutional level, with primary care and emergency transfer settings focusing on initial assessment and transfer indicators, and tertiary hospitals collecting more fine-grained data across the care continuum. Establishing health information exchanges or data lakes with open standards and uniform interfaces could help break down persistent data silos. Multimodal AI and generative models also created new opportunities for integrating structured and unstructured clinical data in acute care workflows (110).

Large-scale, multicenter, and cross-regional data collection efforts were also needed to improve diversity and representativeness in acute and critical care datasets (111). Domain-adversarial training and robustness validation strategies could help models adapt to heterogeneous data from different institutions, regions, and patient populations (112). A nationwide collaborative clinical data network could integrate fragmented resources across prehospital rescue, emergency care, ICU treatment, and rehabilitation follow-up. Standardized templates could capture demographics, dynamic vital signs, laboratory and imaging findings, interventions, and outcomes in a more consistent manner. Dynamic knowledge-update mechanisms were also necessary, particularly in acute and critical care, where diagnostic criteria and treatment protocols evolve rapidly. Version-controlled medical knowledge graphs could continuously track changes in clinical guidance, such as the transition from SIRS-based to SOFA-based sepsis assessment, allowing models to be updated gradually. For novel diseases or unexpected emergencies, emergency learning pipelines and limited-sample adaptation strategies could support more timely model revision.

#### 3.5.4 Improving explainability

Future development was expected to prioritize interpretable models tailored to acute care needs (113). In emergency departments and ICUs, clinicians often needed to make rapid decisions under conditions of uncertainty, and black-box outputs might be difficult to trust or act upon. Approaches such as rule-based systems, decision-tree algorithms, or other interpretable frameworks could improve transparency in time-sensitive settings. Chain-of-thought-style reasoning frameworks and agent-based architectures might also support more traceable decision processes (114), particularly when they were used to clarify how multiple data sources contribute to a recommendation (115). Incorporating explainability constraints during model training might further improve clinician trust and facilitate safer human-AI collaboration in high-stakes environments (106).

#### 3.5.5 Technical infrastructure and adaptive capacity

Effective implementation of AI in acute and critical care required adequate computational infrastructure, stable power supply, and reliable digital systems. Emergency departments and ICUs often depended on rapid, uninterrupted decision support, and insufficient computational capacity could delay model deployment or limit real-time use. Expanding investment in hardware, high-performance computing centers, and secure hospital data systems was therefore important. Training and recruiting interdisciplinary professionals with expertise in both AI and critical care was equally necessary. Joint programs between medical schools and engineering faculties, as well as specialized training in critical care AI, could help cultivate a workforce capable of translating technical innovation into frontline practice.

Model adaptability was also a key technical requirement in acute and critical care, where patient conditions and disease patterns changed quickly. Nationwide disease surveillance networks that integrate multi-tiered medical institutions and IoT-enabled devices could support earlier recognition of emerging clinical trends. Federated learning offered one approach to cross-institutional model development while addressing privacy concerns and data fragmentation by enabling training without direct exchange of raw data (116). Incremental and online learning strategies could also support rapid model updating when novel diseases, new treatment protocols, or changing case mixes emerged.

#### 3.5.6 Ethical and regulatory pathways

Legislation such as the Personal Information Protection Law and the Data Security Law was refined with clauses specific to acute care (117). Dedicated regulations should define the responsibilities of institutions, developers, and data users in high-risk clinical environments. Stronger oversight, access control, and auditing mechanisms were also necessary, together with regular staff training in ICUs and emergency departments.

Error-detection and self-correction capabilities should also be incorporated into AI systems used in acute and critical care. Fine-tuning combined with retrieval-augmented generation could support self-correction and improve reliability in complex clinical scenarios (118). Human-in-the-loop quality control systems could further support continuous improvement by feeding clinically identified errors back to developers. Importantly, AI systems should remain strictly auxiliary tools, with all outputs subject to clinical verification by qualified professionals (119, 120).

**Figure 2:**
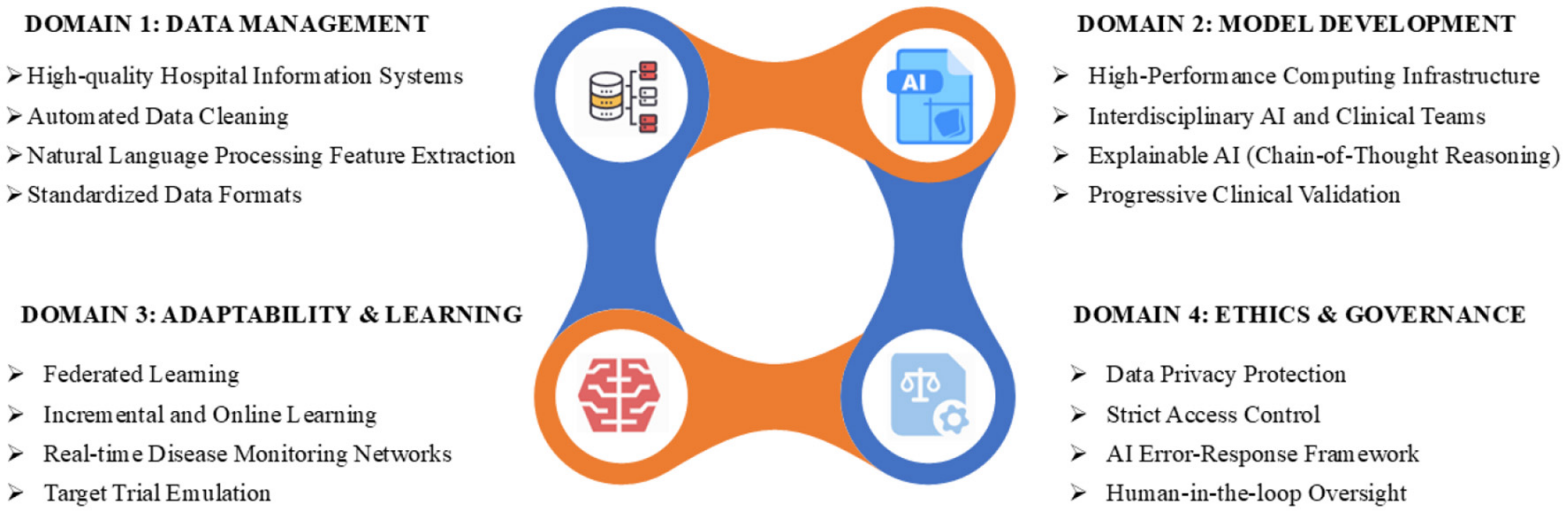
Key strategies for overcoming challenges in medical AI for acute and critical care. HIS, hospital information system; AI, artificial intelligence; CoT, chain-of-thought.

**Figure 3:**
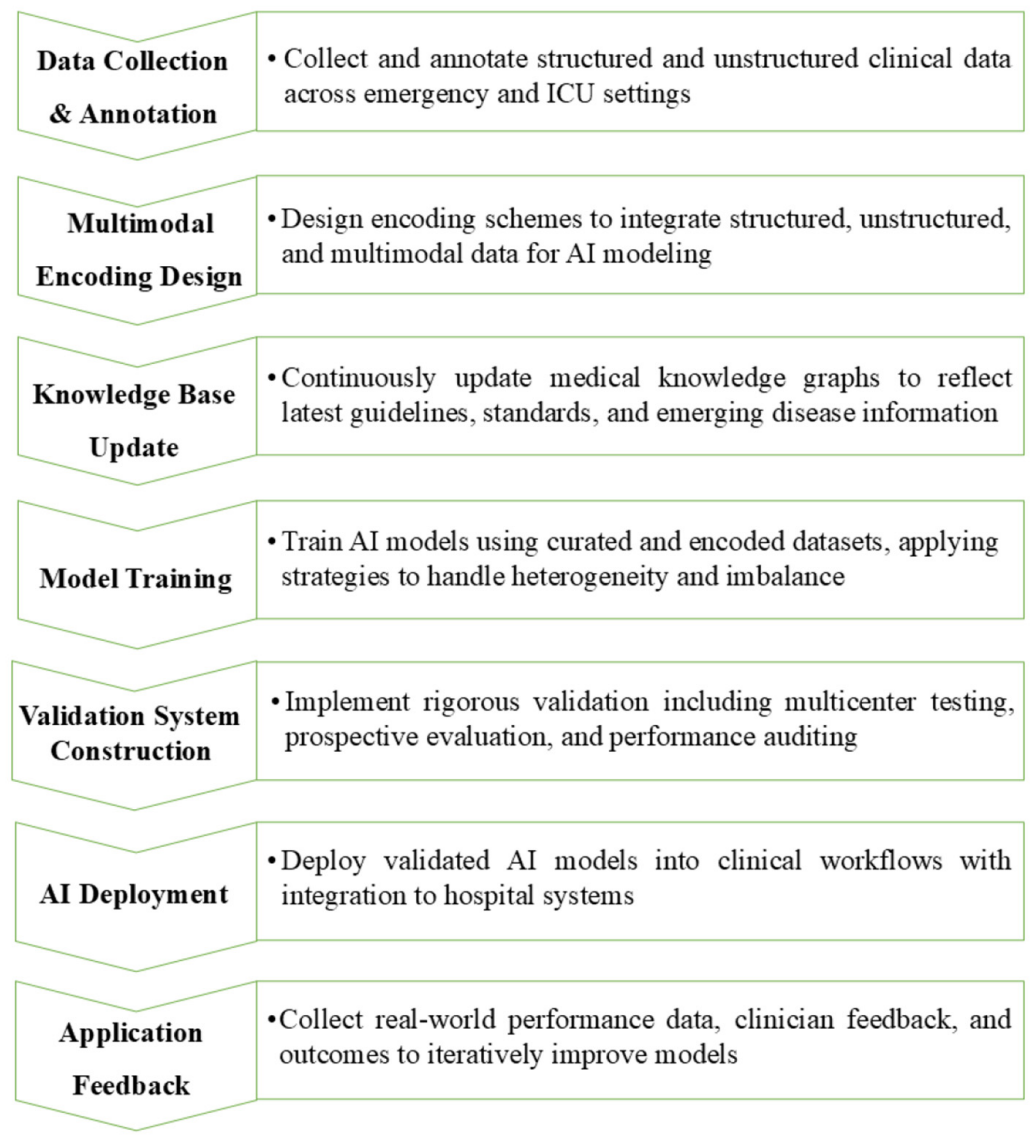
Data-driven workflow for medical AI model training in acute and critical care. AI, artificial intelligence; ICU, intensive care unit.

The review summarized key strategies, including high-quality hospital information systems, automated data cleaning, structured feature extraction, interdisciplinary teams, explainable AI, federated learning, and ethical oversight. Figure 2 illustrates a comprehensive framework for implementing medical AI in acute and critical care. Figure 3 outlines a seven-step workflow: data collection and annotation, multimodal encoding design, dynamic knowledge base updates, model training, closed-loop validation, AI deployment, and real-world feedback. This integrated approach facilitates the development of clinically effective, adaptable, and ethically responsible AI systems.

## 4 Conclusion

Medical AI holds substantial promise for improving decision-making and patient outcomes in acute and critical care. Nevertheless, important challenges remain in data quality, model reliability, clinical integration, prospective validation, and ethical and regulatory oversight. Future development should focus not only on predictive accuracy, but also on trustworthy, explainable, and workflow-integrated systems that can support real-world bedside decision-making in emergency departments and intensive care units. This review outlines key strategies to address these barriers and proposes a general workflow for data-driven AI model development in acute and critical care.
